# Transcriptomic analysis of maternally provisioned cues for phenotypic plasticity in the annual killifish, *Austrofundulus limnaeus*

**DOI:** 10.1186/s13227-017-0069-7

**Published:** 2017-04-21

**Authors:** Amie L. Romney, Jason E. Podrabsky

**Affiliations:** Department of Biology, Portland State University, P.O. Box 751, Portland, OR 97207 USA

**Keywords:** Diapause, Maternal effect, Maternal-to-zygotic transition, Transcriptome, RNA-seq, Alternative splicing

## Abstract

**Background:**

Genotype and environment can interact during development to produce novel adaptive traits that support life in extreme conditions. The development of the annual killifish *Austrofundulus limnaeus* is unique among vertebrates because the embryos have distinct cell movements that separate epiboly from axis formation during early development, can enter into a state of metabolic dormancy known as diapause and can survive extreme environmental conditions. The ability to enter into diapause can be maternally programmed, with young females producing embryos that do not enter into diapause. Alternately, embryos can be programmed to “escape” from diapause and develop directly by both maternal factors and embryonic incubation conditions. Thus, maternally packaged gene products are hypothesized to regulate developmental trajectory and perhaps the other unique developmental characters in this species.

**Results:**

Using high-throughput RNA sequencing, we generated transcriptomic profiles of mRNAs, long non-coding RNAs and small non-coding RNAs (sncRNAs) in 1–2 cell stage embryos of *A. limnaeus*. Transcriptomic analyses suggest maternal programming of embryos through alternatively spliced mRNAs and antisense sncRNAs. Comparison of these results to those of comparable studies on zebrafish and other fishes reveals a surprisingly high abundance of transcripts involved in the cellular response to stress and a relatively lower expression of genes required for rapid transition through the cell cycle.

**Conclusions:**

Maternal programming of developmental trajectory is unlikely accomplished by differential expression of diapause-specific genes. Rather, evidence suggests a role for trajectory-specific splice variants of genes expressed in both phenotypes. In addition, based on comparative studies with zebrafish, the *A. limnaeus* 1–2 cell stage transcriptome is unique in ways that are consistent with their unique life history. These results not only impact our understanding of the genetic mechanisms that regulate entrance into diapause, but also provide insight into the epigenetic regulation of gene expression during development.

**Electronic supplementary material:**

The online version of this article (doi:10.1186/s13227-017-0069-7) contains supplementary material, which is available to authorized users.

## Background

Development in the annual killifish *Austrofundulus limnaeus* is unique for four major reasons. First, the embryos develop slowly for their size and can enter into a state of developmental and metabolic arrest termed diapause at three distinct developmental stages [1, 2]. Second, during early development the cell movements associated with epiboly are separated temporally and spatially from gastrulation and formation of the embryonic axis [3, 4]. Third, embryonic development is plastic and embryos can develop along at least two alternative pathways based on an interaction of maternal programming and incubation environment [5]. Finally, embryos of *A. limnaeus* can tolerate and survive extreme environmental stresses, such as long-term anoxia and dehydration [6]. Despite these unique characters, the development of *A. limnaeus* is quintessentially vertebrate and appears to utilize the same conserved genetic networks that govern development of the typical vertebrate body plan [4]. The mix of unique and apparently conserved developmental characteristics of this species makes it an excellent model for examining the evolutionary and mechanistic adaptations of novelty in vertebrate development.

In all vertebrates, the activation of the embryonic genome is delayed for several to many cell divisions following fertilization [7, 8]. During this time, cellular processes are directed by maternal products (RNA transcripts, proteins, ribosomes and hormones) packaged in the egg during oogenesis [9–12]. In the zebrafish *Danio rerio*, and most other organisms, maternally derived mRNA transcripts direct early gene expression in the zygote through cleavage and blastulation, but are targeted for degradation coincident with activation of embryonic genome transcription [11, 13–16]. This process has been termed the maternal-to-zygotic transition and coincides with what is known as the mid-blastula transition in some vertebrates [15]. While many studies have reported on the existence of the maternal-to-zygotic transition during vertebrate development, very few have actually profiled the contents of newly fertilized eggs to identify maternally contributed gene products [13, 17].

Maternally packaged RNAs underlie cellular programming in vertebrate embryos that ensures proper early development such as cleavage, formation of the blastula and gastrulation [7, 17, 18]. Patterns of early cleavage determine distribution of yolk resources, maternally derived factors, and establish the morphogenetic fields that define the vertebrate body plan. Thus, maternally packaged RNAs and their regulation, modification and stability are especially important during the earliest phases of embryonic development. Understanding the key transcripts that must be packaged into an oocyte, and the mechanisms that determine their stability, translatability and thus their ultimate expression and action could help explain a diversity of developmental phenomena.

There are two major avenues for altered expression of the maternal transcriptome during early development: alternative packaging of transcripts and modifications of stability or translatability to existing transcripts. For example, alternative mRNA splicing has been found in many vertebrate systems as part of cellular responses to environmental stimuli [19, 20]. Alternatively, small non-coding RNAs (sncRNAs) have been implicated in the regulation of gene expression by altering mRNA stability or translation in a variety of contexts including embryonic development [21–26]. The role of sncRNAs in the highly conserved processes of early vertebrate development has received far less attention than other potential mechanisms of regulation. The ability of a single sncRNA to target multiple transcripts and effect global alterations in gene expression makes this an attractive mechanism for control of gene networks in the absence of an active genome. Many recent studies suggest a critical role of regulatory RNAs in early vertebrate development [27].

Annual killifish (Aplocheiloidei) are represented by hundreds of species of small tropical and subtropical fishes in Africa and South America [28]. The annual killifish, *A. limnaeus*, is native to ephemeral ponds on the coast of Venezuela [29–31]. These ponds are short-lived (several weeks to several months), typically small (3–200 m^2^), and offer a harsh developmental environment with large fluctuations in key environmental parameters such as temperature, oxygen partial pressure, pH and water availability [6, 32]. These fish grow rapidly to sexual maturity and spawn continuously during their typically short (a few months) adult life, leaving their embryos to survive the dry season. Importantly, an expanding set of genomic tools, including a draft genome assembly [33], are now available for *A. limnaeus,* making it possible to explore genetic and epigenetic mechanisms during development.

Annual killifish embryos are a unique system for examining developmental physiology because they are capable of entering an endogenously cued metabolic dormancy termed diapause as an adaptive phenotype to survive the seasonal drying of their pond habitats [34, 35]. Diapause can occur at three distinct developmental stages, diapause I, II, III [2]. There are unique physiological traits associated with each stage of diapause; however, diapause II embryos show the greatest degree of tolerance to environmental stresses such as desiccation and anoxia [6, 36]. Entrance into diapause II (from here forward referred to as diapause) is one of two possible trajectories during the embryonic development of annual killifish [34, 35]. While a large proportion of embryos enter diapause as their normal mode of development, others are capable of “escaping” diapause and instead develop continuously until the pre-hatching stage [2]. Early embryos on either trajectory are indistinguishable; however, during somitogenesis the trajectories diverge in both morphological and physiological characters such that the timing of developmental events is unique for each trajectory [5]. The mechanisms that regulate these trajectories are currently unknown, but recent studies suggest possible maternal provisioning during oogenesis or another form of epigenetic programming [5, 37]. Typically, younger females produce almost exclusively escape embryos and older females produce almost exclusively diapausing embryos [5]. Yet, there is a great deal of interindividual variation and some females will consistently produce escape or diapausing embryos independent of age. Interestingly, embryonic incubation temperature can override maternal influences and lead to embryos that develop exclusively along the escape and diapause trajectories. Incubation at 20 °C results in 100% diapausing embryos, while incubation at 30 °C results in 100% escape embryos [5]. Thus, both the pre-fertilization and post-fertilization environment can affect developmental trajectory. Given the harsh conditions in which these embryos exist and the short duration of pond inundation, the developmental trajectory of an embryo will likely have a profound effect on its survival and on the survival of the local population. The mechanisms that mediate this type of critical genome–environment interaction are unknown but critically important for understanding the basic mechanisms of development in the natural world.

The events that occur in early development are thought to be some of the most conserved processes in biology. For example, there is striking conservation of function in the genes that regulate blastulation and gastrulation in all animals [38, 39]. A great deal of work has gone into characterizing these shared molecular pathways, while relatively few studies have focused on gene expression changes that may underlie plasticity during vertebrate development [40]. In fact, the vast majority of gene expression studies on developing vertebrates have focused on systems that exhibit little to no intraspecific plasticity in development [41–43]. Here, we report on the transcriptome of newly fertilized eggs of the annual killifish *A. limnaeus* collected from females that are known to produce 100% escape and 100% diapausing embryos. This paper describes for the first time the maternally derived transcriptome of early embryos of *A. limnaeus*, explores the possibility of maternal control of entrance into diapause through differential packaging of RNA and uses a comparative approach to identify aspects of the transcriptome that may explain some of the unique attributes of development in this species compared to more typical teleosts. Evidence is presented that suggests splice variants of genes common to both trajectories and differential packaging of sncRNAs are both possible routes for maternal control of developmental trajectory. Further, comparative analysis of *A. limnaeus* to zebrafish suggests a unique maternally packaged transcriptome in *A. limnaeus* that is consistent in many ways with the unique developmental patterns observed in annual killifishes.

## Results

### Maternally derived poly-A RNA transcriptome

Details regarding poly-A RNA transcriptome library sequencing and bioinformatics can be found in Additional file 1. RNA-seq methods detected 12,329 transcripts in the 1–2 cell stage transcriptome of *A. limnaeus* with a mean expression of 2 or greater fragments per kilobase of transcript per million mapped reads (FPKM), representing about 60% of all sequences in the libraries (Additional file 2). The 20 most abundant transcripts included nuclear- and mitochondrially encoded protein-coding, ribosomal RNA and long non-coding RNA genes (Table 1). Gene ontology (GO) analysis revealed enrichment for highly expressed genes (500 most abundant transcripts; >180 FPKM) in categories that include RNA-binding proteins, cytoskeletal proteins as well as redox reaction enzymes and pathways enriched for ATP synthesis and G-protein-coupled signaling (Additional file 3).

**Table 1:** Top 20 most abundant mRNA transcripts expressed in 1–2 cell stage embryos of *A. limnaeus*

Gene symbol	Gene description	Gene type	Expression (FPKM)
*COI*	*Cytochrome C oxidase I* ^a^	Protein coding	8491
*16S*	*16* *s ribosomal RNA* ^a^	rRNA	7635
*LOC106526789*	*Ferritin, middle subunit*	Protein coding	5782
*LOC106522651*	*Uncharacterized*	lncRNA	5697
*COIII*	*Cytochrome C oxidase III* ^a^	Protein coding	5017
*LOC106523488*	*Claudin*-*like protein, ZF*-*A89*	Protein coding	4858
*LOC106532313*	*Uncharacterized*	lncRNA	4619
*LOC106518911*	*Uncharacterized*	lncRNA	4331
*LOC106512296*	*Uncharacterized*	lncRNA	4322
*LOC106533547*	*Late histone H2B.L4*-*like*	Protein coding	4219
*LOC106530205*	*Ribosyldihydronicotinamide dehydrogenase [quinone]*	Pseudogene	3774
*LOC106512721*	*Uncharacterized*	lncRNA	3752
*LOC106511212*	*Zinc finger protein 36, C3H1 type*-*like 2*	Protein coding	3561
*LOC106527827*	*Peptidyl*-*prolyl cis*–*trans isomerase*-*like*	Protein coding	3539
*LOC106519960*	*Tubulin alpha chain*-*like*	Protein coding	3403
*ATPase6*	*ATP synthase 6* ^a^	Protein coding	3270
*ubb*	*Ubiquitin B*	Protein coding	3259
*LOC106532441*	*Ribosyldihydronicotinamide dehydrogenase [quinone]*-*like*	Protein coding	3078
*LOC106526526*	*Tumor*-*associated calcium signal transducer 2*-*like*	Protein coding	3042
*hspb1*	*Heat-shock 27*-*kDa protein 1*	Protein coding	3011

*FPKM* Fragments per kilobase of transcript per million mapped reads

^a^Mitochondrially encoded gene

### Differential expression of poly-A RNA

**Fig. 1:**
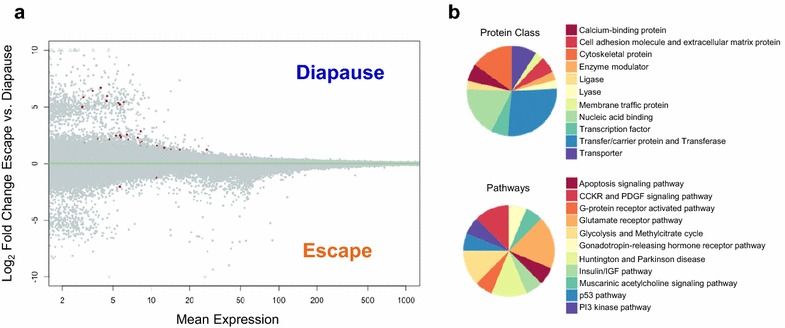
Alternative splicing of poly-A RNA in embryos of *A. limnaeus* that will develop along two alternative developmental trajectories. **a** Differential exon usage in mRNA gene transcripts that are packaged into diapause- and escape-destined 1–2 cell stage embryos of *A. limnaeus*. Of the 57 exons that were significantly different between trajectories (*red symbols*, FDR < 0.1, *t* test) 49 are upregulated in diapause-bound embryos, while only 8 are upregulated in escape-bound embryos. **b** GO term analysis for transcript variants between diapause- and escape-bound embryos of *A. limnaeus* (*P* < 0.05) suggests enrichment for exons expressed in genes for a variety of molecular and metabolic pathways including glycolysis and the insulin/IGF signaling pathway

**Fig. 2:**
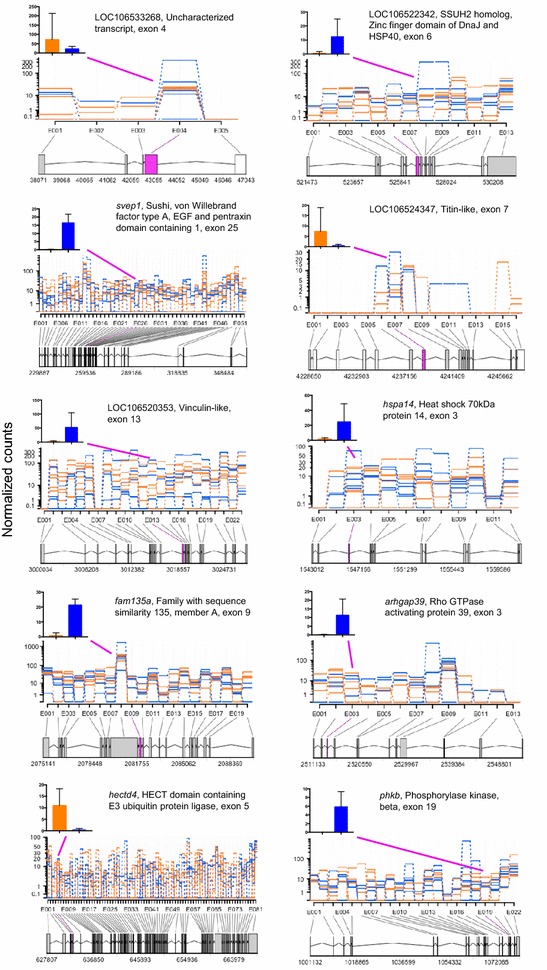
Top 10 genes with developmental trajectory-specific splice variants based on statistical significance. Each biological replicate is graphed separately in the exon usage graphs with *orange lines* indicating escape-bound embryos and *blue lines* indicating diapause-bound embryos. The *x*-axis on the plots indicates the exon number and the mapping location of the exon on the appropriate contig from the *A. limnaeus* genome file. Note that the *y*-axis is a log scale which tends to mask the differential expression of the exons, and thus we have provided a bar graph on a linear scale to better illustrate the mean (±SD) levels of expression for the differentially expressed exon within each gene. *Blue bars* indicate diapause-bound embryos, while *orange bars* represent escape-bound embryos

Gene-level analysis determined that none of the 12,329 genes were differentially packaged in diapause- and escape-destined embryos (*t* test, FDR > 0.10). However, 57 genes showed differential exon usage between diapause- and escape-destined embryos (Fig. 1a). GO analysis suggests that the differentially expressed exons are enriched in a number of pathways including glycolysis and insulin signaling (Fig. 1b). These exons reside within genes with a variety of functions including: heat-shock protein (hsp), *hspa14*; pre-mRNA splicing, *snrnp200*; cytoskeletal proteins, *vinculin*; transmembrane ion transport, *sideroflexin*; cell adhesion, *svep1*; and mTOR signaling of cell growth and proliferation, *ribosome protein s6 kinase.* The 10 genes with the most significant differentially expressed exons (based on FDR adjusted *P* values) are presented in Fig. 2, and the entire list of 57 genes can be found in Additional file 4.

### Comparative transcriptomics

**Fig. 3:**
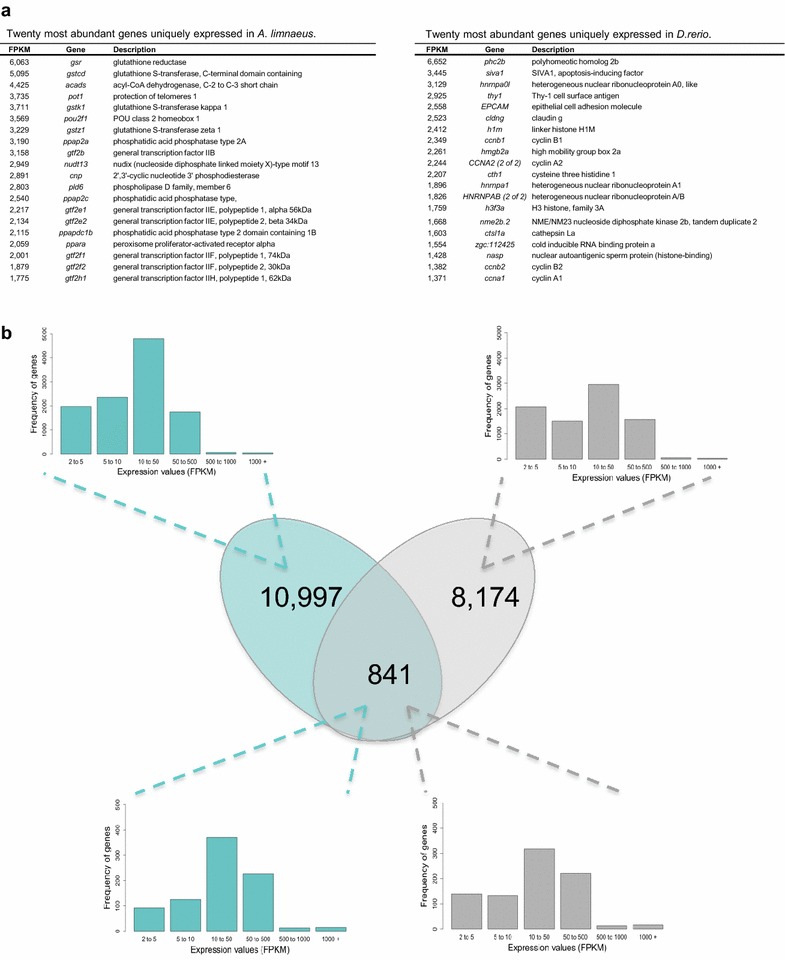
Comparative analysis of poly-A transcriptomes in 1–2 cell stage embryos of *D. rerio* and *A. limnaeus*. **a** The 20 most abundant transcripts and their FPKM values that are unique to the transcriptome of either *A. limnaeus* or *D. rerio*. **b** Venn diagram depicting the number of shared (orthologous) and non-shared transcripts in 1–2 cell stage embryos of *A. limnaeus* (*turquoise*) and *D. rerio* (*gray*). Frequency histograms show the distribution of expression values of shared (orthologous) and non-shared genes and indicate similar patterns of transcript abundance between the two species

**Fig. 4:**
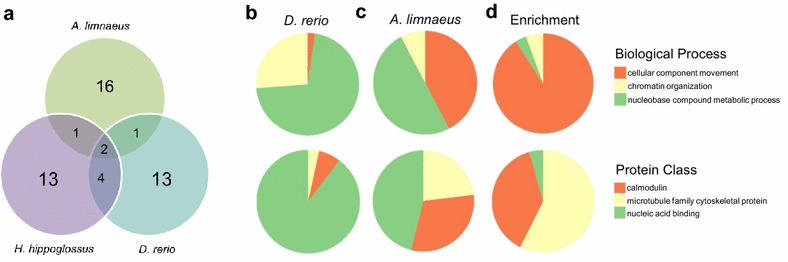
The relationship of the maternally packaged transcriptome of *A. limnaeus* to other teleosts. **a** Venn diagram showing shared numbers of the 20 most abundant transcripts in 3 species of fish with 1–2 cell stage Illumina-sequenced transcriptomes*: A. limnaeus* (this study)*, D. rerio* [17] and *H. hippoglossus* [44]. **b**–**d** Gene ontology analysis comparing the top 100 transcripts in the 1–2 cell stage transcriptomes of *A. limnaeus* and *D. rerio*. Pie charts represent the quantities of GO categories in (**b**) *D. rerio* and **c** *A. limnaeus* for biological process classification (*top*) and protein class (*bottom*). **d** Go terms enriched in the *A. limnaeus* compared to *D. rerio* (*P* < 0.05) transcriptome. For more details see the text and Additional file 5

Comparison of the transcriptome of 1–2 cell stage embryos from *A. limnaeus* to the same stage embryos of *D. rerio* (as reported by Harvey et al. [17]) revealed thousands of species-specific transcripts that likely identify major differences in developmental programs between annual killifish and zebrafish (Fig. 3). Of the 9018 transcripts present in the 1–2 cell stage transcriptome of *D. rerio* (≥2 FPKM, protein-coding only [17]) less than 10% (841) were identified as orthologous to the transcripts expressed in *A. limnaeus* (Fig. 3). Comparatively, 89% (10,997) and 91% (8174) of the expressed transcripts (≥2 FPKM, protein-coding only) were unique to *A. limnaeus* and *D. rerio*, respectively. The group of 841 genes that were expressed in both species shared similar patterns of abundance. However, there were significant differences in expression of these transcripts between the two species, with the most differentially expressed transcripts coming from mitochondrially encoded genes, *claudin* and *ubiquitin*, among others (Table 2). Transcripts encoded in the mitochondrial genome are twofold to fourfold higher in most cases for *D. rerio* compared to *A. limnaeus* (Table 3). While there are only 3 comparable datasets available in the literature for 1–2 cell stage embryos (*Hippoglossus hippoglossus* [44], *D. rerio* [17] and data presented here for *A. limnaeus*), it appears that each species has a unique expression pattern for the 20 most abundant maternally packaged transcripts (Fig. 4a). When comparing the 100 most abundantly expressed transcripts in the *D. rerio* and *A. limnaeus* 1–2 cell stage transcriptomes (Fig. 4b–d), the *A. limnaeus* transcriptome is enriched in GO terms for ion binding and transport as well as cytoskeletal structure and function (Fig. 4d). Alternatively, the *A. limnaeus* transcriptome is underrepresented compared to *D. rerio* in transcripts with GO terms for metabolic processes (Fig. 4d, Additional file 5).

**Table 2:** Most differentially expressed orthologous genes between *A. limnaeus* and *D. rerio*

Gene name	Percent difference (%)	Species dominantly expressed
Mitochondrially encoded ATP synthase 6	61	*D. rerio*
Claudin-4-like/d	58	*D. rerio*
Mitochondrially encoded cytochrome c oxidase subunit II	45	*D. rerio*
Ubiquitin B/C	30	*A. limnaeus*
Mitochondrially encoded cytochrome c oxidase subunit I	23	*D. rerio*
Mitochondrially encoded NADH dehydrogenase 3	23	*D. rerio*
Calmodulin-like/3a	22	*A. limnaeus*
Mid1-interacting protein 1-like	20	*A. limnaeus*
Mitochondrially encoded cytochrome B	20	*D. rerio*
Translation elongation factor 1 alpha 1	19	*D. rerio*
Mitochondrially encoded NADH dehydrogenase 1	18	*D. rerio*
Mitochondrially encoded NADH dehydrogenase 6	17	*D. rerio*
Heat-shock cognate 70-kDa protein/heat-shock protein 8 (hspa8), mRNA	12	*D. rerio*
H3 histone, family 3B/3d	12	*D. rerio*
Mitochondrially encoded NADH dehydrogenase 2	11	*D. rerio*
Mitochondrially encoded cytochrome c oxidase subunit III	10	*D. rerio*
Small ubiquitin-like modifier 3b	10	*D. rerio*
Osteoclast stimulating factor 1	10	*A. limnaeus*
Heterogeneous nuclear ribonucleoprotein A/B	9	*D. rerio*
Ribosomal protein L23	9	*A. limnaeus*
Acidic (leucine-rich) nuclear phosphoprotein 32 family, member B	8	*D. rerio*

Percent difference—difference in ortholog rank-order abundance in either transcriptome, expressed as percent of total transcripts

**Table 3:** Mitochondrial genes of *A. limnaeus* and *D. rerio* and their expression summary in the 1–2 cell stage transcriptome

Gene code	Gene name	*A. limnaeus*		*D. rerio*		*D.r.*/*A.l.*
		Rank	FPKM	Rank	FPKM	Fold difference
*mt*-*co1*	Cytochrome c oxidase subunit 1	1	8491	4	7127	0.8
*mt*-*co3*	Cytochrome c oxidase subunit 3	5	5017	7	6417	1.3
*mt*-*atp6*	ATP synthase subunit a	16	3270	3	9270	2.8
*mt*-*co2*	Cytochrome c oxidase subunit 2	26	2436	5	6798	2.8
*mt*-*nd4*	NADH-ubiquinone oxidoreductase chain 4	28	2309	26	1916	0.8
*mt*-*nd2*	NADH-ubiquinone oxidoreductase chain 2	39	1738	16	2924	1.7
*mt*-*nd1*	NADH-ubiquinone oxidoreductase chain 1	48	1419	12	3208	2.3
*mt*-*cyb*	Cytochrome b	63	1057	14	2991	2.8
*mt*-*nd4* *l*	NADH-ubiquinone oxidoreductase chain 4L	93	755	126	501	0.7
*mt*-*nd6*	NADH-ubiquinone oxidoreductase chain 6	100	714	21	2373	3.3
*mt*-*nd5*	NADH-ubiquinone oxidoreductase chain 5	105	688	47	1197	1.7
*mt*-*nd3*	NADH-ubiquinone oxidoreductase chain 3	106	674	17	2858	4.2
*mt*-*atp8*	ATP synthase protein 8	633	149	89	684	4.6

Rank—rank order of abundance in the transcriptome

FPKM—fragments per kilobase of transcript per million mapped reads

Fold difference—expression value in *D. rerio* divided by the expression value in *A. limnaeus*

### Small RNA transcriptome

**Fig. 5:**
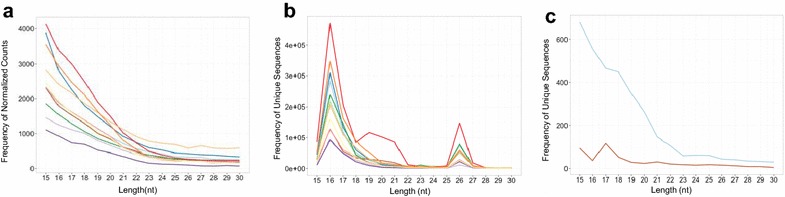
Maternally packaged sncRNA transcriptome of *A. limnaeus*. Frequency distribution of **a** normalized sequence reads and **b** unique sequences as a function of sequence length in the sncRNA libraries (*n* = 12). Each library is a *different color*. **c** There is a high diversity of sncRNA sequences with lengths between 15 and 23 nucleotides that are unknown (*blue line*) compared to those that could be annotated (*red line*) by sequence similarity to those cataloged in RNA databases

Details of the small RNA library sequencing and bioinformatics results can be found in Additional file 1. The *A. limnaeus* 1–2 cell stage sncRNA transcriptome is characterized by 3379 sncRNA transcripts expressed at a level of ≥2 normalized counts per million reads (Additional file 6). To better examine the diversity of sequence reads we grouped only identical sequences, not making any assumptions about mature sequence length, clustering or shared gene origin. At fertilization, embryos possess a wide diversity of sncRNA sequences. The majority of the sequence reads were short in length (<20 nt), while longer sequences (>25 nt) were less abundant (Fig. 5a). However, analysis of unique sncRNA sequence abundance as a function of sequence length revealed two dominating size classes; the highest abundance size class was 16 nt, while the second most abundant was 26 nt (Fig. 5b).

**Fig. 6:**
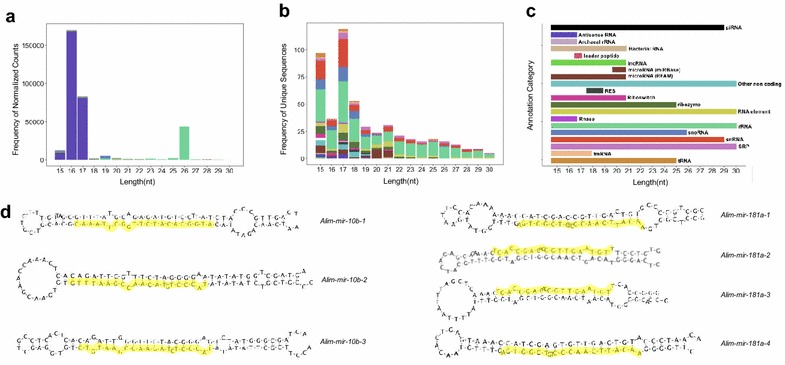
Rfam database annotation of the maternally packaged sncRNA transcriptome of *A. limnaeus.* **a** The most abundant sncRNAs are 16–17 nucleotides in length and annotate as antisense RNAs, while the second most abundant group are sequences that are 26 nucleotides in length that annotate as ribosomal RNA (see panel **c** for a color key to annotation category). **b** The highest diversity of unique sncRNA sequences is in the 15 and 17 nucleotide length categories. Note the enrichment of miRNA sequences in the 20–22 nucleotide range as expected, even though miRNAs are not a dominant part of the sncRNA transcriptome. **c** Some annotation categories have distinct size ranges, while others span the entire range of sizes explored in this study. **d** Putative micro-RNA precursor structures and consensus mature sequences (*highlighted in yellow*) annotated as mir-181a (*Alim*-*mir*-*181a1*-*3*) and mir-10b (*Alim*-*mir*-*10b1*-*4*)

Between 57 and 73% of sncRNA sequence reads in each sample library could be annotated to known RNAs based on sequence alignment with miRBase Release 21 and Rfam version 12.1 [45, 46]. All sncRNAs were also annotated based on sequence alignment to a database of piwiRNA (piRNA) sequences in RNAdp v. 2.0 [47]. Annotations of miRNA sequences were similar in Rfam and miRBase, and the following summary of annotations is based on the Rfam results only. Of the 3379 unique sncRNA sequences, the majority had lengths below 20 nt and were not annotatable, while only 21% (722) annotated to known RNAs (Fig. 5c). The sncRNA sequences with the highest abundance annotated as antisense RNAs (55% of total reads, Fig. 6a) with the remainder including fragments of ribosomal RNAs (<1% of total reads) and small nucleolar RNAs (<1% of total reads; Fig. 6b, c). Surprisingly, miRNA annotations comprised <1% of the sncRNA transcriptome in 1–2 cell stage embryos (Fig. 6a–c). Of the 22 unique mature sequence variants that annotated as miRNAs, consensus sequences were generated for *mir*-*181* and *mir*-*10*. High confidence precursor sequences, based on sequence and secondary structure modeling, were prepared for submission to miRBase for *Alim*-*mir*-*10* and *Alim*-*mir*-*181* (Fig. 6d).

The 3379 unique sncRNA sequences mapped to approximately 33,000 locations in the genome, with 61% mapping to intergenic regions and 39% within exons (Additional file 6). The remaining alignments were positioned within introns (8%) or as a combination of categories (1%). Most sequences (2796) mapped to the genome in multiple locations (2–100). Only 583 mapped to exactly one position, with 51% of these in intergenic regions, 21% in introns, 27% in exons. Interestingly, 41% aligned in an antisense orientation, and 4 sequences mapped to the mitochondrial genome (3 of 4 to the *ND3* gene).

The two most abundant sncRNA sequences had average counts per million reads of 168,769 and 79,972 and annotated as antisense RNAs in Rfam, while the third-most abundant (43,648 counts) annotated as rRNA based on genome alignments (Table 4, Additional file 7). The top twenty most abundantly expressed sncRNAs are dominated by sequences unknown to Rfam. However, they appear to be unique and non-repetitive sequences as determined by a low number of alignments to the genome (1–2 alignments each with perfect match of leftmost 15 bases). However, loosening the parameters just slightly increases the genomic alignments to over 100 locations (Additional file 8).

**Table 4:** Top 20 most abundantly expressed small RNA transcripts expressed across all libraries in 1–2 cell stage embryos of *A. limnaeus*

Sequence	Length^a^	Mean expression^b^	Rfam ID	Class of RNA
*TCAGACAACTCTTAGC*	16	168,769	RF02179	Antisense RNA
*CTCAGACAACTCTTAGC*	17	79,972	RF02179	Antisense RNA
*GTCGCCTGAATACCGCAGCTAGGAAT*	26	43,648	Not annotated	
*GAGCGCCGCGACTCCTCA*	18	12,557	Not annotated	
*GAGCGCTGCGACTCCTCA*	18	10,491	Not annotated	
*TCTCAGACAACTCTTAGC*	18	9808	Not annotated	
*ACGAGAGCTTTGAAGACCGA*	20	7987	Not annotated	
*AACGAGAGCTTTGAAGACCGA*	21	6983	Not annotated	
*CGAGAGCTTTGAAGACCGA*	19	6119	Not annotated	
*CAGACAACTCTTAGC*	15	5280	RF02179	Antisense RNA
*GGAGCGCCGCGACTCCTCA*	19	5198	Not annotated	
*TCAGACAACTCTTAGA*	16	4734	Not annotated	
*GGTCGCCTGAATACCGCAGCTAGGAAT*	27	4638	Not annotated	
*GAGCGCCGCGACTCCT*	16	3836	Not annotated	
*TCAGACAACTCTTAG*	15	3806	RF02179	Antisense RNA
*ATGTCAAAGTGAAGAAATT*	19	3729	Not annotated	
*TCACTCTGCATCTCTC*	16	2158	Not annotated	
*CTCAGACAACTCTTAG*	16	1902	Not annotated	
*AAACGAGAGCTTTGAAGACCGA*	22	1616	Not annotated	
*GGAGCGCCGCGACTCCT*	17	1528	Not annotated	

^a^Length in base pairs

^b^Expression in normalized counts per million reads

### Differential expression of sncRNAs

Only four sncRNA sequences (≥2 normalized counts) were differentially expressed between embryos on the escape and diapause trajectories (Table 5). These four sequences do not match any known RNA sequences in Rfam or miRBase. The most differentially expressed gene sequence, *ACAACGTGTGATACA*, aligned once to the genome in an intronic region of a gene predicted to be *zinc finger protein 646*-like (*LOC106517361*; Additional file 8). The other differentially expressed sequences had two alignment positions in the genome (using strict parameters aligning the left-most 15 nt with 0 mismatches). Exploring the role of these sncRNAs is critical to understanding their possible involvement in post-transcriptional modification of other components of the 1–2 cell stage transcriptome.

**Table 5:** Differentially expressed sncRNAs in diapause- and escape-bound embryos of *A. limnaeus*

Sequence	Bp^a^	Exp.^b^	Log_2_FC^c^	Sig.^d^	Pheno.^e^	15 bp^f^	12 bp^f^	10 bp^f^	8 bp^f^	Alignment^g^	Orientation^h^	Position^i^
ACAACGTGTGATACA	15	26.9	5.1	0.008	Escape	1	23	103	183	LOC106517361 (Zinc finger protein 646-like)	Antisense	Intragenic, intronic
TAGTATATAGGACTA	15	46	4.1	0.036	Escape	2	13	60	179	LOC106525548 (neurexin-2-like)/unannotated region	Antisense/sense	Intragenic, intronic/Intergenic
GGCTCTGAATACATTAG	17	5.8	4.5	0.036	Escape	2	126	131	159	LOC106520853 (RNA-binding motif, single-stranded-interacting protein 3)/LOC106533203 (endosialin-like)	Antisense/antisense	Intragenic, intronic/Intragenic, exonic
TCGGAACTCACCCAGTC	17	15.8	4	0.044	Diapause	2	4	6	6	Unannotated region/LOC106512419 (uncharacterized LOC106512419)	Sense/antisense	Intergenic/Intragenic, exonic

^a^Length of the sncRNA in base pairs

^b^Mean counts per million mapped reads across all samples

^c^Log_2_ fold change

^d^Statistical significance (FDR < 0.1)

^e^Developmental phenotype with the higher abundance of the sncRNA

^f^Number of alignment locations in the *A. limnaeus* genome for the sequence if the number of bases indicated is used starting at the 5’ end of the sequence

^g^The locations in the *A. limnaeus* genome where the 15-bp sequence aligned

^h^The orientation of each alignment location for the 15-bp sequence in reference to the *A. limnaeus* genome annotation

^i^Position within the alignment location that the sncRNA aligned to

## Discussion

This study demonstrates for the first time a profile of maternally packaged RNAs for *A. limnaeus*. Due to the critical nature of early developmental events across multiple timescales (individual life span to evolutionary time) it is highly likely that each of the transcripts identified in this study plays an essential role in supporting early development and diapause in this species. This work serves as a foundation of information from which to build working hypotheses concerning the maternal control of the unique developmental attributes of annual killifishes.

### General patterns in the poly-A RNA transcriptome

Perhaps not surprisingly, the protein-coding transcriptome of 1–2 cell stage embryos from *A. limnaeus* shares many similarities with fertilized eggs from other teleosts when analyzed on a presence–absence basis [17, 43, 44, 48, 49] and GO analysis indicates similar function between the transcriptomes of *A. limnaeus* and *D. rerio*. Early vertebrate development is thought to be highly constrained and conserved, and thus, it is reasonable to hypothesize that similar gene expression patterns would be required in all species. However, for the 3 species for which data are directly comparable (same developmental stage and sequencing methodology) each species appears to have a unique pattern of highly abundant transcripts with only 2 of the top 20 transcripts shared across all three species. In a direct comparison of *A. limnaeus* and *D. rerio*, each species expresses an order of magnitude more transcripts that are unique, compared to those that are shared. This species-specific expression in the maternally derived transcriptome likely reflects the different developmental strategies and environments of each species.

### Stress tolerance

Early development, specifically the cleavage stages, tends to be sensitive to environmental stress. It is certainly the case in *A. limnaeus* that stress tolerance is lowest during the first 4 days of development [6, 50, 51]. It has been proposed that overexpression of stress tolerance genes may have a negative effect on development in some species. For example, heat shock that induces HSP expression in zebrafish embryos leads to a high proportion of abnormal embryos [52]. Also, overexpression of the molecular chaperone HSP 70 in *Drosophila* larvae increased thermal tolerance, but also caused a notable decrease in developmental rate and had other negative effects [53, 54]. In contrast, reduction of two small molecular weight chaperones, *p26* and *artemin*, appears to slow development in diapause-bound embryos of *Artemia* [55, 56]. In other studies, inhibition of HSP 90 led to teratogenic effects due to an unmasking of cryptic variation in *Drosophila* and zebrafish [57–59]. Thus, buffering the effects of environmental stresses is likely an essential component of development, but may affect other processes such as developmental rate or phenotypic expression. The transcriptome of *A. limnaeus* has a number of characters that suggest an increased ability to mitigate the potentially negative impacts of environmental stress. A better understanding of these characters may shed light on how embryos can evolve to tolerate high levels of environmental stress while preserving essential processes required for normal development.

The small molecular chaperone, *hsp 27* (*hspb1*), is one of the top 20 expressed transcripts (>3000 FPKM) in *A. limnaeus* and is represented at a substantially higher level than in zebrafish (2.1 FPKM; Harvey et al. [17]) and was not detected in Atlantic halibut [44]. In another report, in situ hybridization did not detect *hspb1* in zebrafish embryos until the gastrula stage [60]. In a study of *Fundulus heteroclitus*, Tingaud-Sequiera et al. [42] identified the transcriptome of pooled embryonic stages (including the 2–4 cell stage) during exposure to air and addressed the expression of larger inducible heat-shock molecular chaperones such as 40-, 70- and 90-kDa classes, but made no mention of any small heat-shock proteins. In zebrafish, HSP 27 possesses the ability to prevent protein precipitation and is induced under heat stress in post-blastula stage embryos [61, 62]. Further, small hsps have been shown to play a critical role in the survival of heat stress in adult desert fishes [63, 64] and are highly differentially expressed in response to fluctuating daily temperatures in adult *A. limnaeus* [65]. Thus, the high abundance of *hspb1* in the *A. limnaeus* 1–2 cell stage transcriptome appears to be unique and may contribute to their ability to survive stresses imposed by their harsh developmental environment.

Protection from reactive oxygen species is essential for normal cell function and is thought to be critical in mediating survival of oxygen deprivation [66, 67]. Embryos of *A. limnaeus* are the most anoxia-tolerant vertebrates [6, 36, 51] and can develop normally even under extreme hypoxia [68]. Thus, it may not be surprising that maternal provisioning of antioxidant systems would be elevated in this species. Some of the most highly expressed unique sequences in the *A. limnaeus* transcriptome are involved with glutathione metabolism, a key mechanism to deal with oxidative stress [69]. In addition, transcripts for the superoxide dismutase genes (*sod1 and sod2*) are packaged at a much higher level in embryos of *A. limnaeus* (863 and 46 FPKM) compared to *D. rerio* (87 and 19 FPKM). Maternal packaging of *sod1* is thought to protect embryos from oxidative damage during development, when metabolic demands are high [70]. However, the low metabolic rate of early *A. limnaeus* embryos may suggest other reasons for maternal packaging of *sod1* that are more consistent with survival of environmental stress, perhaps playing a role in their unique tolerance of oxygen deprivation.

All organisms possess cellular mechanisms that help maintain homeostasis in the face of environmental stress; many of these mechanisms are activated in the cellular stress response (CSR). A minimal stress proteome (MSP) was described by Kultz [71] that included 44 proteins and protein families that participate in the CSR. Analysis of the 1–2 cell stage transcriptomes from *A. limnaeus* and *D. rerio* suggests a similar number of genes represented in the MSP that are maternally packaged (Table 6). These transcripts represent 2% or less of the transcriptome and less than 1% of the most abundant 25% of transcripts. Based on this admittedly limited evaluation, neither species appears to be particularly enriched for potentially stress-responsive gene expression. However, the nature of the most abundant transcripts from this list is different in the two species. While mitochondrial genes critical for metabolism are highly abundant in both species, they dominate the most abundant transcripts in *D. rerio*, while in *A. limnaeus* the two most highly abundant MSP genes are a molecular chaperone and a thioredoxin (Additional file 9). These data suggest some potentially important functional differences in the stress-responsive transcripts that are maternally packaged in these two species that warrant future attention.

**Table 6:** Number of genes designated in the minimal stress proteome (or MSP) identified in the *A. limnaeus* and *D. rerio* transcriptomes at the 1–2 cell stage

Abundance rank	Number of MSP genes		% of transcriptome	
	*A. limnaeus*	*D. rerio*	*A. limnaeus*	*D. rerio*
Top 1% of transcripts	12	8	0.10	0.09
Top 5% of transcripts	20	15	0.16	0.17
Top 25% of transcripts	73	62	0.60	0.68
Top 50% of transcripts	128	116	1.05	1.28
Top 75% of transcripts	163	159	1.33	1.75
Total	188	198	1.54	2.18

Abundance rank—transcript abundance as a percentage of the total transcriptome

### Slow developmental rate

A relatively slow developmental rate is a general character of all Atherinid fishes including the cyprinodonts [72]. Even within the Aplocheiloid fishes (annual killifish and their relatives), embryos of annual killifish tend to develop much more slowly compared to closely related non-annual relatives. Recently, rates of cleavage were found to be significantly slower in annual killifish compared to non-annual killifish in three lineages that are thought to have independently evolved the annual life history [73]. Thus, when compared to rapidly developing species such as zebrafish, the transcriptome of 1–2 cell stage embryos of *A. limnaeus* may provide clues to the molecular underpinnings of slow development in Atherinids and specifically for annual killifishes.

A simple comparison of the *D. rerio* and *A. limnaeus* transcriptomes suggests a higher abundance of cell-cycle-associated transcripts in zebrafish. For example, of the top 20 unique and highly abundant transcripts in zebrafish, there are 4 cyclins, 2 histones and a number of other transcripts that are expected to be high in proliferating cells. In contrast, these transcripts are not found among the top 20 unique and highly abundant transcripts in *A. limnaeus*. In addition, 9 of the 20 most differentially expressed transcripts between *D. rerio* and *A. limnaeus* are mitochondrially encoded and are statistically more abundant in *D. rerio*. While these transcripts may be maternally provisioned, it has also been demonstrated that active transcription of the mitochondrial genome occurs very early in development and well before activation of the nuclear genome [74]. Thus, the observed differences in mitochondrially derived transcripts could arise either through maternal provisioning, differences in the rates of mitochondrial transcription or perhaps both. While the abundance rank of these transcripts is high in both *D. rerio* and *A. limnaeus*, they are generally 2–4 times more abundant in the *D. rerio* transcriptome. This higher representation of mitochondrially encoded transcripts is consistent with a more active transcription of the mitochondrial genome in *D. rerio*, compared to *A. limnaeus,* and this may perhaps lead to higher rates of mitochondrial activity.

One remarkable difference between embryos of either species is in the profile of transcripts encoding for zinc finger proteins. A total of 462 zinc finger protein transcripts were present in *A. limnaeus* compared to 165 in *D. rerio*. Many of the genes in both datasets corresponded to counts less than 100 FPKM. However, the RNA-binding protein, *zinc finger protein 36*, *C3H1 type*-*like 2* (*LOC106511212* or *Zfp36l2* in other species), was particularly high in *A. limnaeus* with a gene count of approximately 3500 FPKM, while in *D. rerio*, the paralogs of this gene (*zfp36l1* and zfp36l2) have counts of 8 and 36 FPKM, respectively. The gene *zfp36l2* is shared among all vertebrates and is known to promote mRNA decay through interactions with the 3’-untranslated region (UTR) [75]. This protein has been described as part of a common mechanism that can induce cellular quiescence through post-transcriptional regulation of mRNA stability. Recently, it has been shown that the mRNAs degraded through this mechanism code for proteins that promote cell-cycle progression into the S-phase [76]. The high level of expression for this particular transcript in *A. limnaeus* could be a mechanism for slowing progression through the cell cycle during development. It is also reasonable to hypothesize that this transcript could play a critical role in the regulation of developmental arrest associated with diapause in this species.

The homeobox transcription factor, *nanog,* is known to play a role in the maintenance of pluripotency in a number of animal models [77]. However, in the Medaka, *nanog* acts instead as a critical regulator of cell proliferation during early development that supports cell-cycle progression into the S-phase [77]. Transcripts for *nanog* are abundant (1011 FPKM) in fertilized embryos of zebrafish [17]. When the protein sequence for *D. rerio nanog* (AEZ64150.1) was compared to protein sequences of *A. limnaeus* (NCBI BLASTp) the best resulting match was *LOC106530841,* which annotated as *homeobox protein DLX*-*1*-*like* (36% identity, *e* value = 3e^−31^). Interestingly, expression of this transcript in *A. limnaeus* is much lower (317 FPKM) than that of *nanog* in zebrafish. The apparent altered role for *nanog* in fish development and the lower abundance in embryos of *A. limnaeus* point to another potential mechanism that could slow cell-cycle progression and potentially contribute to the ability of these embryos to arrest development in diapause.

Evolutionary biologists have been discussing the potential importance of developmental rate and conducting laboratory selection experiments to alter this rate for decades [78–82]. However, mechanistic studies that can explain differences in developmental rate are lacking with only a few studies focusing on heterozygosity in a variety of metabolic enzyme isoforms [83–85]. Thus, the differences pointed out here between *A. limnaeus* and *D. rerio* may be the first attempt to evaluate, on a global level, the potential cellular mechanisms for altering developmental rate. We do not have enough data at this point to infer causality, but this study certainly points to a number of likely candidates for future evaluation.

### Regulation of developmental trajectory through alternative mRNA splice variants

There are 57 exons that are differentially expressed in the two alternative developmental trajectories in *A. limnaeus*. These exons are found in genes with a myriad of functions including DNA/RNA binding, protein degradation, intracellular signaling, post-transcriptional and post-translational modifications, the cellular stress response, cytoskeletal and transport properties, as well as cellular adhesion. Most of the exons (86%) are more abundant in diapause-destined embryos and absent or only rarely expressed in escape-bound embryos. It is important to note that the presence or absence of an exon changes not the gene dosage, but rather likely alters the regulation or activity of the gene product. Given the wide range of cellular pathways in which these gene products function, changes in their regulation could have profound effects on cellular and organismal physiology. GO analysis indicates an enrichment in genes with roles in a number of signaling pathways that are known to regulate rates of cell proliferation. It is especially interesting that the insulin/IGF pathway is enriched, given its known role in regulating diapause in *C. elegans* and arthropods [86–88]. This may indicate a role for IGF signaling in the regulation of diapause in *A. limnaeus* which would suggest a perhaps universal role for IGF signaling during animal diapause. It is also interesting that genes important for glycolysis are represented because diapausing embryos of *A. limnaeus* are known to be poised for anaerobic metabolism compared to escape embryos [89]. A role for alternative splicing in the regulation of fish physiology is supported by previous work on estrogen receptors in killifish exposed to estrogenic compounds [19].

While it is beyond the scope of this paper to explore every gene that is represented in this list, one of the mRNA splice variants highly packaged in diapause-bound embryos (over 60-fold higher than in escape-bound embryos) is found within the gene *phkb* (*phosphorylase kinase, beta subunit*). A recent study of this protein suggests it is a modulator of *hsp 27* [90], one of the most abundant transcripts in the *A. limnaeus* 1–2 cell stage transcriptome. In the milkfish, expression of this gene was reduced following exposure to thermal and salinity stress [91]. In the Medaka, *phkb* is a key regulator of glycogen synthesis and contributes to calcium and insulin signaling pathways [92]. Given the noted importance of insulin signaling to the regulation of diapause across a variety of animal species, this transcript holds exceptional promise as a target for future functional studies and suggests a potential avenue for alteration of developmental trajectory by incubation temperature.

The discovery of these differentially packaged mRNA variants suggests that alternative splicing rather than differential gene expression may be critical for determining maternal influence on developmental trajectory in *A. limnaeus*. This is a novel result and suggests a subtle role for changes in gene regulation in the control of diapause in this species. Future studies will be required to test for the influence and function of these genes in response to temperature and other factors that are known to affect developmental trajectory.

### The small non-coding RNA transcriptome

A high diversity of sncRNAs, such as demonstrated here, is common in early developmental stages of animals and is likely important for the proper regulation of gametogenesis and fertilization [93, 94]. To our knowledge this is the first report of the sncRNA transcriptome of a 1–2 cell stage fish embryo. The profiles of abundance, diversity and sequence length distribution presented here for *A. limnaeus* are similar to those reported for the zebrafish 256-cell stage embryo [41]. However, it is worthy of note that few mature miRNA sequences were identified here in *A. limnaeus*, while a great variety of miRNAs are present in zebrafish at the 256-cell stage and in later developmental stages of *A. limnaeus* (Romney and Podrabsky, unpublished observations). This may indicate that mature miRNAs are not a common or major component of the maternally packaged transcriptome in fish embryos.

In *C. elegans*, miRNAs are known to be key regulators of the developmental switch associated with entrance into the diapause-like dauer state [95]. Recent studies suggest diapause-specific miRNAs in flesh flies as well [96]. In addition, miRNAs are critical for reactivation of delayed implanting mouse embryos [97, 98]. The lack of miRNA diversity in 1–2 cell stage embryos of *A. limnaeus*, and the fact that no miRNA transcripts appear to be differentially expressed in association with either developmental trajectory suggest that maternal control over entrance into diapause is not likely mediated through any currently described miRNA. It is possible that some of the novel sequences discussed below may act as miRNAs, but more work is needed to explore this possibility.

The high diversity of sncRNAs identified in embryos of *A. limnaeus* falls into two basic categories: those having many sequence variants with low abundance and those with few sequence variants that are highly abundant. While a functional role for the low abundance sequences cannot be ruled out, it is also possible that these are the products of RNA degradation [99]. However, it is important to note that degradation is a perfectly acceptable means to generate biologically active molecules and thus these sequences should not be summarily dismissed. However, given the large number of sequences involved, we have chosen to focus on the highly abundant transcripts that have few variants. We propose a role for these RNAs similar to sncRNA such as endogenous small interfering RNAs (endo-siRNAs) or regulatory genes derived from longer RNAs such as tRNAs, rRNAs and small nucleolar RNAs (snoRNAs). Understanding the role of these potentially novel sncRNAs could elucidate gene regulatory pathways that support alternative developmental trajectories in this species.

The most abundant sncRNA sequences annotated as antisense RNAs, specifically the ST7 antisense RNA 1 conserved region 1 (RF02179). In humans, this long non-coding RNA is involved in the cellular response to DNA damage [100] and is described as a tumor suppressor gene [101, 102], but there is a paucity of information available. When aligned in an antisense orientation in the *A. limnaeus* genome these sequences map to RNA genes, zinc finger proteins, as well as a gene annotated as synaptophysin like (involved in synaptic vesicular transport). When mapped in a sense direction, the sequences align to regions in rRNA genes suggesting they could be generated from excision of internal spacer RNAs from rRNA transcripts. Small RNAs derived from rRNAs and tRNAs have recently been described as important regulators of cell differentiation [93, 99]. It is particularly interesting that these sncRNA sequences may target zinc finger proteins, which appear to be enriched in the transcriptome of *A. limnaeus*. Obviously, the complete picture of the significance of these sncRNAs cannot be determined by expression profile alone, and functional studies are needed to draw additional conclusions.

Only 4 sncRNAs were packaged into embryos at statistically different levels in embryos developing on the two developmental trajectories. All of these sequences are novel, and thus, we currently do not know enough about their biology to make valid predictions for their mode of action or potential targets. However, if we make the assumption they are acting as antisense RNAs to block translation or induce degradation of mRNA targets, a cohesive story emerges that is consistent with a role for these sncRNAs in the regulation of cell proliferation in escape embryos and the potential for chromatin remodeling control of gene expression in diapause-bound embryos. For instance, three of the potential target sequences for sncRNAs that are more abundant in the escape-bound embryos are mRNA transcripts that encode for proteins known to regulate cell-cycle progression. One sequence is antisense to an intron in a zinc finger protein 646-like sequence that contains a SFP1 domain. SFP1 in yeast is a known transcriptional repressor that regulates ribosomal protein expression and blocks the G_2_/M transition of the cell cycle [103, 104]. Another sequence could specifically target two *A. limnaeus* genes. One target, RNA-binding motif, single-stranded-interacting protein 3 (RBMS3), is known to bind the c-Myc promotor and reduce cell proliferation through alteration of β-catenin expression in at least two types of human cancer [105, 106]. Another target for this sncRNA could be the transcript for endosialin-1, also known as TEM-1 in humans. In humans and mice, high TEM-1 expression is associated with pericyte proliferation and angiogenesis and acts through platelet-derived growth factor receptor pathways that lead to activation of immediate early genes like *c*-*fos* [107, 108]. The function of endosialin-1 is currently not understood during early development and has not been characterized in *A. limnaeus* or any other fish. Reduction of this protein could reduce proliferation if it acts as it does in mammals. However, what we know about this protein is restricted to its role in pericyte cells of adult mammals, and it is possible that a context-dependent role for endosialin-1 during early development could be critical for other essential functions such as cell migration or differentiation. The important aspect of this protein is that it contains the functional domains necessary to interact with cell proliferation signaling pathways and thus has the potential for regulation of cell proliferation or perhaps migration. Another potential targeted mRNA transcript in escape-bound embryos codes for a neurexin-2-like protein. The transcript for this protein is maternally packaged in *Xenopus* and is highly expressed during early development in zebrafish, although its function is unknown [109, 110], and thus the role that this protein may play in determination of developmental trajectory in *A. limnaeus* remains unclear. The one sncRNA sequence that is upregulated in diapause-bound embryos targets an uncharacterized protein with SANT and reverse transcriptase domains. Proteins with SANT domains are known to be highly expressed in proliferating cells especially during early development and have a role in the regulation of chromatin organization through histone acetylation [111, 112]. Thus, reduction of this protein could have large-scale effects on gene expression. Unfortunately, we do not know when or where in the developing embryo that these sncRNAs may be active, and future studies to inhibit or modify their expression are needed to evaluate their potential role in altering developmental trajectory in *A. limnaeus*.

## Conclusion

This study reports for the first time the maternally packaged transcriptome of an annual killifish and represents one of only a handful of similar studies on early vertebrate embryos. The data support a transcriptomic poise that could support the unique characters of development in *A. limnaeus* compared to other fishes. Surprisingly, the transcriptomes of *A. limnaeus* and *D. rerio* are more unique than similar, even at the 1–2 cell stage. Unlike the transcriptomes of other teleost species, *A. limnaeus* embryos contain an abundance of gene transcripts that support the slow developmental rates and stress tolerance specific to their life history. For the first time, splice variants of mRNAs are identified that are differentially packaged into oocytes in a vertebrate with alternative developmental trajectories. In addition, the sncRNAs that are differentially packaged suggest an important mechanism for post-transcriptional control that may contribute to the regulation of developmental trajectory, and support the unique embryonic characters in this lineage. Studies such as this are essential to broaden our understanding of the evolution of developmental and phenotypic plasticity.

## Methods

### Animals

Adult *A. limnaeus* were housed in the PSU aquatic vertebrate facility and cared for according to standard laboratory methods established for this species [113] that were approved by the PSU Institutional Animal Care and Use Committee (PSU Protocol #33). This laboratory strain of *A. limnaeus* was originally collected near the town of Quisiro in Venezuela [32] and has been cultured continuously in the laboratory since 1995. Embryos were collected twice a week from 42 pairs of fish during controlled spawning events. Clutches of embryos (20–200 embryos/clutch) were kept separate by spawning date and mating pair. Approximately 2 h after each spawning event (1–2 cell stage), all but 10–15 embryos from each clutch were flash-frozen in liquid nitrogen. The remaining embryos were used to determine the proportion of diapause and escape embryos produced by that spawning pair on that date. These embryos were transferred into embryo medium and maintained at 25 °C in darkness [113] for 17–21 days post-fertilization (dpf) when they were scored as escape- or diapause-bound embryos using previously established criteria [5]. Total RNA samples were prepared from pools of embryos (30–50 embryos per sample) representing 6 females producing either 100% diapausing or escape embryos (*n* = 6 pooled samples for each trajectory, see Additional file 1 for details).

### RNA extraction

Total RNA was extracted from whole embryos using TRIzol reagent (Life Technologies; 50 µl/embryo) according to the manufacturer’s instructions for tissues containing polysaccharides. RNA concentration and purity were assessed by UV absorbance at 260 nm and calculating A_260_/A_280_ ratios (Tecan Infinite M200 Pro with NanoQuant plate, Switzerland). RNA integrity was assessed by agarose gel electrophoresis. RNA samples were maintained at −80 °C until use.

### Poly-A RNA sequencing

cDNA libraries were prepared using the Illumina TruSeq RNA Sample Preparation Kit (v2, Illumina, San Diego, CA, USA) following the manufacturer’s instructions with 1 µg of total RNA as starting material. The purified cDNA libraries were quantified by qPCR and their quality confirmed on a 2100 Bioanalyzer (Agilent Technologies, Santa Clara, CA, USA) using a DNA 1000 chip. The libraries were sequenced (100 nt paired-end reads, 4 samples multiplexed per lane on the flow cell) on an Illumina HiSeq 2000 at the Oregon Health & Science University (OHSU).

### sncRNA sequencing

sncRNA libraries were prepared using the Illumina TruSeq small RNA sample preparation kit following the manufacturer’s instructions with 1 µg of total RNA as starting material. The RNA samples used to prepare these libraries are the same samples used to prepare the poly-A RNA-sequencing libraries. Eleven cycles of PCR amplification were used for library production. Libraries were quantified by qPCR and quality checked on an Agilent Bioanalyzer 2100 using a DNA 1000 chip. The libraries were sequenced (100 cycles, paired-end, 12 samples multiplexed per flow cell lane) at OHSU using an Illumina HiSeq 2000.

### Bioinformatics pipeline

A variety of software packages were used to process and analyze the transcriptomic data. CLC genomics workbench (version 6.5) for a Mac Pro (2 × 3.06 GHz 6-Core Intel Xeon and 64 GB RAM) was used for genomic mapping and generation of count data for the sncRNA libraries. The rest of the analyses were performed in a UNIX environment on the Portland State University computing cluster (Dell PowerEdge R730; 2× Intel(R) Xeon(R) CPU E5-2695 v3 with 28 cores @ 2.30 GHz and 256 GB RAM). Differential gene expression and exon usage analyses were performed using the R Bioconductor packages DESeq2 and DEXSeq [114, 115]. Gene ontologies were assigned using software tools on the PANTHER Web site [116].

### Analysis of poly-A RNA sequence data

Sequence quality was initially assessed using FastQC, version 0.10.1, [117] to ensure high-quality data. Sequence reads were filtered on quality scores and trimmed for the presence of adapter sequences using Trimmomatic [118] with the settings “ILLUMINACLIP:2:30:7:1:true,” “SLIDINGWINDOW:5:15,” “LEADING:20,” “TRAILING:20” and “MINLEN: 25.” Quality reads were mapped to the *A. limnaeus* genome 1.0 using the *very fast local* preset in Bowtie2 [119]. Preserved paired reads after trimming were aligned in paired-end mode, and any orphaned mates after trimming were aligned in single-end mode. Reads that aligned to the *A. limnaeus* genome with 0 mismatches were used for expression analyses. Gene counts (union mode) were generated for all samples using the *summarizeOverlaps* function of the GenomicAlignments package from Bioconductor [120] and the NCBI *A. limnaeus* genome annotation Release 100 [33]. Count matrices were filtered for genes with 1 or more normalized counts summed across all replicates. Ontologies of overrepresented genes were determined using PANTHER software with Bonferroni-corrected *P* value <0.01 [116].

Gene abundance (FPKM) and differential expression analysis were performed using DESeq2 in the R Bioconductor package. Differential gene expression between diapause- and escape-destined embryos was determined on gene count data using the negative binomial distribution and estimations of mean–variance dependence [114]. Differential exon usage was tested using DEXSeq [115]. In both cases, differential expression was evaluated using a Benjamini–Hochberg multiple comparisons adjusted FDR of 10%.

### Analysis of sncRNA sequence data

Sequence files were preliminarily examined using FastQC, version 0.10.1 [117], to explore sequencing quality. Small RNA reads were trimmed for quality and adapters with Trimmomatic using the settings “ILLUMINACLIP:2:30:7:1:true,” “SLIDINGWINDOW:5:15,” “LEADING:20,” “TRAILING:20” and “MINLEN: 15.” Trimmed reads that aligned to the *A. limnaeus* genome with 0 mismatches were retained and counted using the “annotate and merge” function in CLC workbench (version 6.5; CLCbio, Arhus, Denmark). Read counts were normalized across all libraries by DESeq2 to generate a catalog of small RNAs per treatment (average of 2 counts per million or greater) as well as differential expression based on a log_2_ fold change of 1 or greater using the Benjamini–Hochberg multiple comparisons adjusted FDR of 10%.

Sequences were annotated by alignment to small RNA databases allowing up to two mismatches to sequences for *Danio rerio, Fugu rubripes, Tetraodon nigroviridis* and *Oryzias latipes* using miRBase (v.21), Rfam (v. 12.1) and to all *D. rerio* piRNAs downloaded from Ensembl (Zebrafish V10). Small RNA sequences were mapped to the *A. limnaeus* genome using Bowtie and evaluated for proximity to annotated genes. For those sncRNA sequences annotated as miRNAs, consensus genomic sequences were determined by alignment to the *A. limnaeus* genome with perfect matches. Precursor stem-loop secondary structures were predicted from expanded regions of 80–100 nucleotides upstream in the 5′ direction using the Vienna package RNAfold prediction tool in Geneious software (R 8.1.6).

### Comparative transcriptomics

Orthologous transcript pairs between *A. limnaeus* and *D.* rerio were identified by using NCBI BLASTn to align all RNA sequences of one species to the other species and vice versa. Transcript pairs were determined as reciprocal best hits (RBH) when the sequence alignment between two transcripts resulted in the best matching score for both comparisons. The resultant list was cross-referenced to the maternally packaged transcripts dataset of Harvey et al. for *D. rerio* [17], and thus gene names for both species are based on the zebrafish annotation for this analysis.

## Additional files

**Additional file 1.** Details of RNA samples and the resulting cDNA sequencing libraries prepared from diapause- and escape-bound embryos.

**Additional file 2.** The poly-A transcriptome of *A. limnaeus* 1–2 cell stage embryos.

**Additional file 3.** GO analysis of transcriptome of *A. limnaeus* in 1–2 cell stage embryos.

**Additional file 4.** Differentially expressed exons in 1-2 cell stage embryos of *A. limnaeus* (FDR *P* value < 0.10).

**Additional file 5.** GO analysis of top 100 most abundant transcripts in the 1–2 cell stage transcriptomes of *A. limnaeus* and *D. rerio*.

**Additional file 6.** Alignment information of sncRNAs for *A. limnaeus*, regarding the nearest gene and its position within the gene or genomic region.

**Additional file 7.** Most abundant sncRNAs in *A. limnaeus* 1–2 cell stage transcriptome and the annotations of their genomic alignments.

**Additional file 8.** Summary of genome alignments for most abundantly expressed sncRNA in the *A. limnaeus* 1–2 cell stage transcriptome.

**Additional file 9.** Number of genes designated in the minimal stress proteome identified in the *A. limnaeus* 1–2 cell stage transcriptome.

## Abbreviations

cDNA: complementary DNA
FPKM: fragments per kilobase of transcript per million mapped reads
GO: gene ontology
lncRNA: long non-coding RNA
miRNA: micro-RNA
mRNA: messenger RNA
PCR: polymerase chain reaction
piRNA: piwiRNA
qPCR: quantitative polymerase chain reaction
rRNA: ribosomal RNA
sncRNA: small non-coding RNA
snRNA: small nuclear RNA
snoRNAs: small nucleolar RNA
tRNA: transfer RNA
